# Knowledge, attitude and practice study of HIV in female adolescents presenting for contraceptive services in a rural health district in the north-east of Namibia

**DOI:** 10.4102/phcfm.v4i1.342

**Published:** 2012-07-24

**Authors:** Alexis Ntumba, Vera Scott, Ehimario Igumbor

**Affiliations:** 1 School of Public Health, University of the Western Cape, South Africa

## Abstract

**Background:**

Namibia bears a large burden of Human Immunodeficiency Virus (HIV), and the youth are disproportionately affected.

**Objectives:**

To explore the current knowledge, attitudes and behaviour of female adolescents attending family planning to HIV prevention.

**Methods:**

A cross-sectional study design was used on a sample 251 unmarried female adolescents aged from 13 years to 19 years accessing primary care services for contraception using an interviewer-administered questionnaire. Data were analysed using Epi Info 2002. Crude associations were assessed using cross-tabulations of knowledge, attitude and behaviour scores against demographic variables. Chi-square tests and odds ratios were used to assess associations from the cross-tabulations. All *p-*values < 0.05 were considered statistically significant.

**Results:**

A quarter of sexually active teenagers attending the family-planning services did not have adequate knowledge of HIV prevention strategies. Less than a quarter (23.9%) always used a condom. Most respondents (83.3%) started sexual intercourse when older than 16 years, but only 38.6% used a condom at their sexual debut. The older the girls were at sexual debut, the more likely they were to use a condom for the event (8% did so at age 13 years and 100% at age 19 years).

**Conclusions:**

Knowledge of condom use as an HIV prevention strategy did not translate into consistent condom use. One alternate approach in family-planning facilities may be to encourage condom use as a dual protection method. Delayed onset of sexual activity and consistent use of condoms should be encouraged amongst schoolchildren, in the school setting.

## Abstrait

**Étude des connaissances, des attitudes et des pratiques relatives au VIH chez les jeunes adolescentes se présentant en vue d'obtenir des services de contraception dans un district de santé rural dans le Nord-est de la Namibie**

**Présentation:**

Le virus de l'immunodéficience humaine (HIV) pèse lourdement sur la Namibie, et les jeunes en sont disproportionnellement affectés.

**Objectifs:**

Étudier les connaissances, les attitudes et les comportements actuels quant à la prévention du VIH des jeunes adolescentes se présentant au service de planification familiale.

**Méthode:**

Un modèle d’étude transversale a été utilisé sur un échantillon de 251 adolescentes non mariées âgées de 13 à 19 ans se présentant dans les services de soins de santé primaires afin de recevoir une contraception, en utilisant un questionnaire administré par des enquêteurs. Les données ont été analysées par Epi Info 2002. Les associations brutes ont été évaluées en utilisant des tableaux à double entrée des résultats des connaissances, des attitudes et des comportements par rapport aux variables démographiques. Des tests du chi-carré et des rapports de cotes ont été utilisés afin d’évaluer les associations générées par les tableaux à double entrée. Toutes les valeurs *p* < 0.05 ont été considérées comme statistiquement significatives.

**Résultats:**

Un quart des adolescentes sexuellement actives se présentant aux services de planification familiale ne disposaient pas de connaissances adéquates sur les stratégies de prévention du VIH. Moins d'un quart (23.9%) d'entre elles utilisait systématiquement un préservatif. La plupart des adolescentes interrogées (83.3%) sont devenues sexuellement actives à plus de 16 ans, mais seulement 38.6% d'entre elles avaient utilisé un préservatif lors de leur premier rapport sexuel. Plus les filles étaient âgées lors de leur premier rapport sexuel, plus elles étaient susceptibles d'utiliser un préservatif lors de ce premier rapport (8% < 0.05 des adolescentes ayant eu leur premier rapport à 13 ans avaient utilisé un préservatif, contre 100% de celles qui avaient eu ce premier rapport à 19 ans).

**Conclusion:**

La connaissance de l'utilisation du préservatif comme stratégie de prévention du VIH ne se traduisait pas par une utilisation régulière du préservatif. Une approche alternative dans les structures de planification familiale pourrait consister à encourager l'utilisation du préservatif comme double méthode de protection. Le report du premier rapport sexuel et une utilisation régulière du préservatif devraient être encouragés chez les élèves, en milieu scolaire.

## Introduction

As in many sub-Saharan countries, Namibia bears a large burden of Human Immunodeficiency Virus (HIV), and the youth are disproportionately affected. Of 14,100 new infections per annum, 44% are amongst youth between 14 years and 24 years of age and 77% in females.^1^ HIV prevalence in the 20–24-year age group is 12.5%, and it is 22.8% in the 25–29-year age group.^2^ The rural district HIV prevalence is 19.2%, whilst nationally the prevalence in 20–24 and 25–29-year-olds in rural areas is 12.4% and 22.1% respectively, and 12.6% and 23.55 respectively in urban areas.^2^ A study in Namibia^3^ found that 60% of adolescents aged 16–19 years were already sexually active and that 6% of girls and 12% of boys stated that they had started having sex before the age of 15. Youth have been targeted in the Namibian government's HIV prevention programme through school-based HIV education programmes4 which operate in rural as well as urban settings. Youth also have access to HIV Counselling and Testing (HCT) centres, which have been established at all primary care facilities and which run outreach community mobilisation activities.

This study was conducted in a rural setting in a health district in the north-east of Namibia where staff working in family-planning clinics reported that adolescent girls were not using condoms. Why were teenage girls demonstrating responsibility for one aspect of sexual and reproductive health (using contraception to protect themselves against unwanted pregnancy), whilst not protecting themselves adequately against HIV? A better understanding of the knowledge, attitudes and behaviour of teenagers accessing family-planning clinics is required to inform appropriate interventions in the health services. The justification for integrated services is overwhelmingly strong in countries with severe generalised HIV epidemics,^4^ and family-planning services are well placed to implement HIV prevention strategies amongst sexually active female adolescents.

### Study setting

The study was carried out in a rural health sub-district in the north-east of Namibia which had one district hospital with eight feeder clinics. The latter were staffed by nurses and HCT and family- planning services were offered at all these sites.

### Aim of the study

This study set out to describe the level of knowledge, attitude and behaviour related to HIV and AIDS of female adolescents accessing primary health services for contraception in a rural health district, and to determine the significance of the association between knowledge and behaviour, if any.

### Contribution to field

Family-planning services are a useful gauge of whether the health services are offering integrated youth services, yet not much research has been done in this setting. The study was designed to respond to reports from ground-level staff indicating that adolescent girls, who seemed to be taking responsibility to protect themselves against unwanted pregnancy (one element of risk) were, however, not using condoms to protect themselves from HIV (another element of risk). It was necessary to carry out research to see whether there was evidence to support these anecdotal claims and to identify underlying knowledge and attitudinal factors towards HIV and AIDS prevention methods to guide programme development, implementation and quality improvement processes.

## Methods

### Design

A quantitative, descriptive cross-sectional study design was used. The study population consisted of unmarried female adolescents aged 13–19 years who were accessing primary health care services for contraception. The district hospital was purposefully selected as its caseload was approximately double that of the individual clinics. Four of the eight health facilities were then selected by simple random sampling. In the second stage of sampling, participants within these five selected facilities were sampled systematically: every second female adolescent on contraception was selected during the 8-week study period until the desired sample size was achieved.

### Sample size

Sample size was calculated using the Stat Calc function in Epi Info 6. It was estimated that a total of 1500 adolescent girls access health facilities for contraception in the district, and that only 25% are using condoms regularly (worst estimate 20%). A minimum sample size of 242 was required to have a 95% confidence level. In this study a total of 251 female adolescents who were accessing the Primary Health Care (PHC) services for contraception were selected, proportional to the total number of adolescents at each facility (40–60 from each facility [Table 1]). All female adolescents who gave consent and signed the consent forms were included in the study until the desired sample was achieved.

**TABLE 1:** Number of participants recruited per facility.

Facility	Patients
District hospital	60
Clinic A	51
Clinic B	46
Clinic C	42
Clinic D	52

### Procedure

An interviewer-administered questionnaire was used to collect data for 8 weeks. Out of the 68 questions from the Family Health International (FHI) standardised questionnaire,^5^ 34 relevant questions were selected; the others were omitted as they were related to marriage, sexually transmitted diseases other than HIV, commercial partners and some background characteristics not relevant to the study. Only one question was added (Question 35: Do you think that a person can get infected with HIV and AIDS by unprotected sexual intercourse?) for the purpose of the study. The questionnaire was pre-tested and found to be reliable.

### Analysing

Data were captured in Excel spreadsheet and imported into Epi Info 2002 (Centers for Disease Control, 2002) for analysis. The same program was used for data analysis. Crude associations were assessed using cross-tabulations of knowledge, attitude and behaviour scores against demographic variables. Chi-square tests and odds ratios were used to assess associations from cross-tabulations, with *p* values < 0.05 considered statistically significant.

To determine whether participants had adequate knowledge of prevention strategies, a composite score was developed based on four questions. Each correct answer awarded one mark: 1. Knowledge of a source to obtain condoms; 2. Knowledge that one can protect oneself from HIV every time one has sex; 3. Knowledge that HIV can be prevented by abstinence; and 4. Knowledge that HIV can be prevented by having one uninfected faithful partner.

All those who scored 3 or 4 were considered as having adequate knowledge of prevention strategies and those who scored 2 or less were considered to have inadequate knowledge. To determine whether participants practiced risky behaviour, two questions were used, each carrying one mark: 1. Consistency in condom use in the last 12 months preceding the survey; 2. Condom use at last sexual intercourse. Those who scored 2 were considered as having low risky behaviour, whilst those who scored 1 and below were considered as having high risky behaviour.

Variables were categorised in the comparative part of the analysis. Current education level was categorised into ‘Grade 8 and below’ and ‘At least Grade 9’. Age was also categorised into ‘16 years and below’ and ‘at least 16 years’.

## Ethical considerations

### Potential benefits and hazards

The participants were not exposed to any physical risks. As a study was dealing with adolescents, obtaining consent from their parents was considered; however, this would have resulted in breaking the confidentiality between the adolescent on contraception and the health service. Some parents might not know that their children were on family planning and this forced disclosure could be harmful to the adolescents and prevent their ongoing access to family planning if the parents objected. In Namibia parental consent is not required for adolescents to be on contraception. It was therefore decided to obtain consent from the adolescents and not the parents.

This decision was supported by the High Degree Committee of the University of the Western Cape, South Africa, and the Namibian Ministry of Health and Social Services Committee.

All participants benefited from health education on HIV transmission and ways to reduce risk. The importance of condom use was promoted and their use demonstrated.

Given the sensitive nature of the content of the questionnaire, psychological stress was anticipated and counselling support was organised. However, none of the participants reacted with any stress so this support was not used. An open-door policy for counselling by nurses at the facilities was put in place should stress arise any time after the interviews.

### Recruitment procedures and consent and confidentiality

Adolescents attending the health services during the study period were approached and invited to participate. The voluntary nature of participation was stressed, and participants were also informed that they had the right not to take part and could withdraw any time they wished without explanation, and that this would not affect the care they received from the health facility. Informed consent was obtained from the participants. To ensure confidentiality and anonymity respondents did not state their names, and after answering each questionnaire was placed in a box.

## Trustworthiness

### Generalisability

The results are generalisable to female adolescents on contraception in the study setting, and the findings could be transferable to similar settings in Namibia and possibly to other low-income countries in Southern Africa where there is a low education level and health services are constrained. The results of the study are not generalisable to youth in general, since this study had a specific focus on those who demonstrated initiative and responsibility in preventing unwanted pregnancy.

### Validity

The design is suited to the research question. The questionnaire used is based on a standardised questionnaire developed by FHI (2000) for unmarried youth (15–19 years) which has been validated across a range of sub-population groups surveyed internationally. Simple random sampling of health facilities and systematic sampling of adolescents (selection bias) took place.

To avoid instrument bias, the questionnaire was translated into the local language (Mbukushu) by a voluntary counselling and testing counsellor who holds an HIV diploma and is fluent in both the local language and English. The interviewers were nurses and community counsellors from participating health facilities, all fluent in English and the local language. They were trained on the interviewer-administered questionnaire and all showed understanding and readiness before data collection began.

## Results

A review of service delivery statistics at all the facilities during January 2007 to November 2007 was carried out. This showed that the district hospital attended to 250 clients under 20 years of age for family planning, whilst the clinics attended to between 114 and 167 over this time period.

**FIGURE 1:**
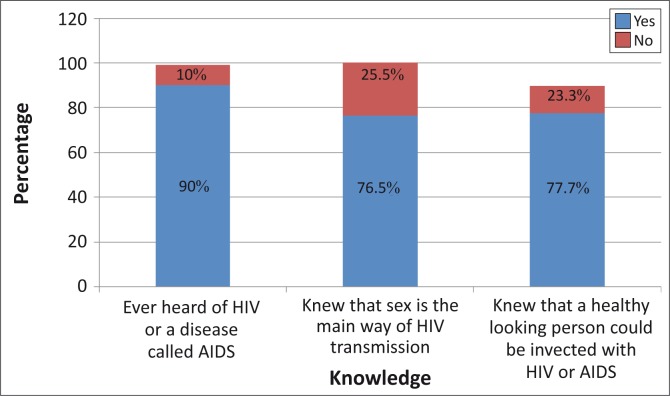
General knowledge about Human Immunodeficiency Virus (*n* = 251).

**FIGURE 2:**
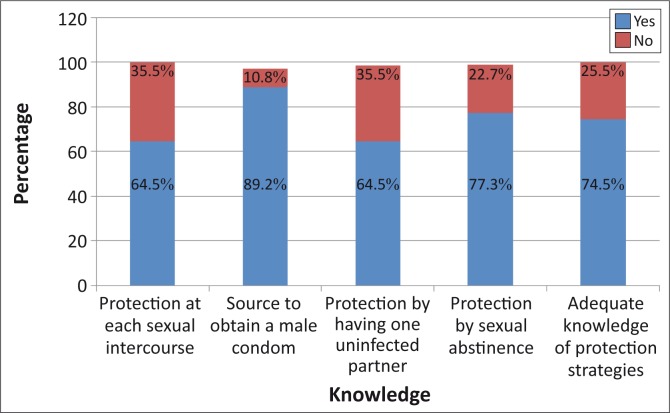
Knowledge of Human Immunodeficiency Virus and AIDS prevention strategies (*n* = 251).

Figure 1 shows levels of general HIV knowledge, whilst Figure 2 shows levels of knowledge of HIV prevention strategies (which were lower). Using the composite score based on the questions related to knowledge of HIV prevention strategies, 74.5% of respondents had adequate HIV prevention knowledge and 25.5% did not.

This study revealed that the majority of respondents (83.3%) started sexual intercourse when older than 16 years, but only 38.6% used a condom at their sexual debut. The older the girls were at sexual debut, the more likely they were to use a condom for the event (from 8% at age 13 years to 100% at age 19 years; Table 2).

**TABLE 2:** Age and condom use at sexual debut (*n* = 251).

Age at sexual debut	*n*	%
11	1	0
12	6	50
13	25	8
14	53	19
15	67	45
16	57	44
17	28	50
18	8	87.5
19	6	100

*n*, Givcen as number participants.

In the 12 months preceding the survey the majority of the respondents (84.8%; *n* = 213) had sexual intercourse. Less than a quarter (23.9%) of these had always used a condom, a few respondents (9.6%) used a condom almost every time, almost half used a condom sometimes (46.5%), and 20.7% never used a condom. The study revealed that less than half of the sexually active respondents (47.3%) had used a condom the last time they had sexual intercourse. Twenty-four per cent of respondents had a low behaviour risk based on their composite score for questions related to risky behaviour.

There was a statistically significant association between age and having heard of HIV (Table 3), and also between education level and having heard of HIV, knowing that a healthy person can have HIV and having adequate knowledge of HIV prevention strategies (Table 4).

**TABLE 3:** Association of Human Immunodeficiency Virus general knowledge, knowledge of prevention strategies and prevention practice with age.

Knowledge	≤ 16 years		> 16 years		*p-*value
	
	%	CI%	%	CI%	
Heard of HIV and AIDs	84.70	76.0–91.2	93.50	88.3–96.8	0.0273
Knows that a healthy-looking person can have HIV	75.50	65.8–83.6	79.10	71.8–85.2	0.1374
Knows that sexual intercourse is the main way of getting HIV	75.50	65.8–83.6	77.10	69.6–83.5	0.2064
Has adequate knowledge of HIV prevention strategies	73.50	51.1–71.5	75.20	72.9–86.2	0.7643
Used a condom at last sexual intercourse	43.90	33.9–54.3	50.00	41.8–58.2	0.688
Is consistent in condom use	25.50	17.2–35.3	22.20	15.9–29.6	0.9757

CI, Confidence Interval.

**TABLE 4:** Association of HIV general knowledge, knowledge of HIV prevention strategies and HIV revention practice with current educational level.

Knowledge	≤ Grade 8		> Grade 8		*p-*value
	
	%	CI%	%	CI%	
Heard of HIV and AIDS	81.50	76.0–91.2	97.40	88.3–99.8	0.0029
Knows that a healthy-looking person can have HIV	74.10	69.7–82.9	85.70	75.9–92.6	0.001
Knows that sexual intercourse is the main way of getting HIV	74.10	69.7–82.9	81.80	71.4–89.7	0.1736
Has adequate knowledge of HIV prevention strategies	70.70	65.7–76.0	83.10	77.8–85.3	0.0374
Used a condom at last sexual intercourse	48.60	42.5–58.1	45.50	34.1–57.2	0.6726
Is consistent in condom use	25	18.7–32.3	22.10	13.4–33	0.9204

CI, Confidence Interval.

There was no significant difference between those who knew that sex was the main way of HIV transmission and those who did not know with regard to consistency of condom use (*p* = 0.0927; Tables 5 and Table 6).

**TABLE 5:** Association of respondents’ knowledge and practice.

Knowledge	Consistent condom use		Inconsistent condom use		*p-*value
	
	%	CI%	%	CI%	
Has adequate HIV general knowledge	25.50	17.2–35.3	74.50	69.4–82.8	0.0927
Has adequate knowledge of HIV prevention strategies	24.10	18.1–30.8	76	72.9–86.1	0.5535

CI, Confidence Interval.

**TABLE 6:** Association of respondents’ knowledge and practice.

Knowledge	Used condom at last sexual intercourse		Did not use condom at last sexual intercourse		*p-*value
	
	%	CI%	%	CI%	
Has adequate HIV general knowledge	73.10	64.2–80.8	26.90	19.2–35.8	0.132
Has adequate knowledge of HIV prevention strategies	79.00	70.6–85.9	21.00	14.1–29.4	0.2116

CI, Confidence Interval.

Only 25.5% of those who knew that sex was the main way of transmitting HIV were consistent in condom use. Knowledge of HIV prevention strategies did not translate into appropriate behaviour, as only a quarter (24.1%; 95% CI 18.1–30.8%) of respondents who had adequate knowledge of HIV and AIDS prevention strategies were consistent (always using them) in condom use.

## Discussion

This study confirmed the low consistent use of condoms amongst adolescents accessing family-planning services in a rural health district in Namibia, despite the adolescents demonstrating adequate general knowledge of HIV and its transmission through unprotected intercourse. Whilst there was some evidence that knowledge supports behaviour (e.g, the association between knowledge that sex is the main way of transmitting HIV and consistency in condom use), on most parameters there seemed to be a gap between knowledge and behaviour. This finding is consistent with that in Petiffor et al.'s earlier study set in South Africa,^6^ in which only 29% of respondents who knew that condom use could protect HIV transmission were consistent in condom use. Cletan and Waltins^7^ have observed that AIDS control programmes face similar challenges to those of the family-planning movement in 1980: 'they know but they do not change.’

A study in a rural province of South Africa^8^ describes some of the reasons why condoms are not used consistently: perceived and real physical side-effects, including reduced pleasure; distrust in the efficacy of condoms; gender-related reasons; and trust in relationships. Dual protection, defined as any strategy that prevents both unwanted pregnancy and sexually transmitted infections, including HIV, has long been promoted as an important preventive approach in reproductive health.^9, 10^ In the family-planning setting, promotion of condoms as dual protection might facilitate condom use.

In this study a significant difference was found between age of sexual debut and use of condoms at sexual debut, with increasing age making the use of condoms more likely. This supports the public health message of delayed sexual debut being an important strategy to reduce risky sexual behaviour in youth.^6, 11^

Stoneburner and Low-Beer^12^ found that substantial HIV reductions in Uganda resulted from public health interventions that triggered a social process of risk avoidance. In Eastern Zimbabwe delayed sexual debut has been associated with a reduction in HIV prevalence in younger cohorts.^13^

In a setting with such a high prevalence of HIV, where integrated services are desirable,^4^ it is disappointing that a quarter of sexually active teenagers attending family-planning services did not have adequate knowledge of HIV prevention strategies. To our knowledge no other studies have been published that assess the extent of HIV prevention knowledge in a general or family- planning setting in Namibia. It is, however, noteworthy that this clinic-based prevalence of HIV prevention knowledge is lower than the national community-based prevalence in rural girls, as measured in the Demographic and Health Survey 2006–2007.^14^ This may be due to the fact that the study setting is a deep rural one with particularly poor socio-economic indicators.

The prevention of HIV and prevention of unwanted pregnancies are two out of the four prevention of mother-to-child-transmission strategies that should be implemented in family-planning settings,^15^ and there would be efficiency benefits through access to target programme recipients. In an integrated approach all female adolescents who attend the primary care services for contraception should be targeted for HIV prevention strategies, with promotion of condoms as protection and life skills development. This is a key policy recommendation which should appeal to policy makers as there are obvious efficiency and effectiveness gains. Policy makers should also be cognizant of the global trend to promote adolescent-friendly services.^4, 16^

A wide variety of service delivery models have been identified for adolescent health services,^17^ ranging from general primary care services to specialised adolescent health centres, to school or community-based services. A framework for the desirable characteristics of youth- or adolescent-friendly services, as set out in a World Health Organization technical paper,^18^ is based on services being accessible, acceptable, appropriate and effective for adolescents. A study in Kenya and Zimbabwe^19^ found that what adolescents value most is confidentiality, short waiting time, low cost and friendly staff. Characteristics such as being a youth-only service, having youth involvement and young staff were rated as less important, suggesting that general or family-planning services based in general primary care settings (such as our study setting) are acceptable to youth. Setting adolescent-friendly standards has been shown to improve the quality of care for adolescents in clinics.^20, 21^ Further training may be required to re-orientate and motivate family-planning health workers on HIV and AIDS prevention. It is not enough that education programmes offer accurate, comprehensive information – they must be tailored to the developmental needs and social contexts of adolescents, and specifically seek to build skills for negotiating sexual behaviours.^16, 22^

In our study, higher education level is also associated with improved HIV general knowledge and greater knowledge of HIV protective strategies. This may be related to the school-based HIV prevention programme called ‘My future is my choice’^4^ which is conducted in the afternoons in secondary school from Grade 8. Given the young sexual debut and the fact that 53% of the population do not reach secondary school,^23^ it may be worthwhile considering whether to introduce the programme to primary schools too. Experience in Burkina Faso, Ghana, Malawi and Uganda has suggested that school-based sex education has strong potential to reach adolescents under the age of 15 years.^24^ Higher education level may also be associated with improved knowledge of protective strategies, because it is one of the broader social determinants of sexual behaviour. Others are poverty, unemployment, and social norms.^16^ These social risks must be addressed together with individual-based risk to be effective in HIV prevention.

### Limitations of the study

Some limitations were noted in this study, including the fact that 12.7% of respondents stated that they had not had sexual partners in the 12 months preceding the survey. It might be that some were using contraceptives to regulate their periods. Alternatively, it might be that some were sexually active but were denying it because they could be embarrassed by the questions and by the interviewers, who they may have known. This situation would affect the validity of the results.

### Recommendations

All female adolescents who attend the primary care services should be targeted for comprehensive HIV education, emphasising prevention strategies and life skills development. All health facilities should also provide condoms to all female adolescents as part of a dual contraception strategy at their monthly visit in the district.

Supervision and mentorship of health workers should include attention to creating adolescent-friendly attitudes which are welcoming and respect confidentiality. Health workers must be supported in developing the necessary knowledge and skills to tailor age-appropriate HIV and AIDS prevention education and couple information with skills development to empower adolescents to negotiate safer sex and condom use.

## Conclusions

Sexually active teenagers attending family-planning services in rural Namibia would benefit from the promotion of dual protection within an adolescent-friendly service which is accessible, acceptable and appropriate for adolescents. Delayed onset of sexual activity and consistent use of condoms should be encouraged amongst schoolchildren, in the school setting.
